# CD40L adjuvant for DNA/MVA vaccine: enhanced protection from acquisition of neutralization sensitive & neutralization resistant mucosal SIV infections

**DOI:** 10.1186/1742-4690-9-S2-P365

**Published:** 2012-09-13

**Authors:** S Kwa, S Sadagopal, J Hong, S Gangadhara, R Basu, L Lai, S Iyer, K Araki, PL Earl, L Wyatt, F Villinger, B Moss, R Ahmed, RR Amara

**Affiliations:** 1 Emory University, Atlanta, GA, USA; 2 NIH, Bethesda, MD, USA

## Background

Generating highly functional antibodies against HIV-1 is critical to prevent infection. Here we evaluated the ability of CD40L (a co-stimulatory molecule for B cells and dendritic cells) as an adjuvant to prevent mucosal infection from neutralization-susceptible (SIVE660) and neutralization-resistant (SIV251) SIVs.

## Methods

Groups of rhesus macaques (10-15 animals/group) were immunized intramuscularly at 0 and 8 weeks with 3mg of DNA/SIV or DNA/SIV-CD40L, and boosted with 10^8^ pfu of MVA/SIV at 16 and 24 weeks. In addition, animals challenged with SIV251 received 10^6^ pfu of MVA/CD40L premixed with MVA/SIV immunizations. Both the DNA and MVA immunogens expressed SIV239 Gag, Pol and Env. At about 24 weeks after final vaccination, animals were challenged intrarectally with either SIV251 (8 weekly doses) or SIVE660 (12 weekly doses) until all unvaccinated controls became infected.

## Results

Adjuvanting DNA with CD40L enhanced (p<0.05) the titer and avidity of antibodies against SIV239 and SIVE660 Envs. Adjuvanting both DNA and MVA with CD40L enhanced (p<0.05) the frequency of B cell follicles with germinal centers (sites of antibody affinity maturation) in the lymph nodes. Following challenge, animals vaccinated with CD40L adjuvant showed markedly enhanced protection from virus acquisition against both SIVE660 (number of challenges for 50% infection was 2 in controls, 5 in DNA/MVA and 12 in DNA/MVA with CD40L; p=0.003, 76% vaccine efficacy) and SIV251 (number of challenges for 50% infection was 2 in controls, 2 in DNA/MVA and 6 in DNA/MVA with CD40L; p=0.04, 55% vaccine efficacy). The enhanced protection against SIVE660 correlated directly with increased avidity of anti-SIVE660 Env antibody and correlation analyses for protection against SIV251 are ongoing. CD40L-adjuvanted animals also showed 100-fold reduction in peak virus replication in both infection models.

## Conclusion

These results demonstrate that CD40L enhances the functional quality of anti-Env antibody and protection from acquisition of neutralization sensitive and resistant mucosal SIV infections.

